# Endocrine and non-endocrine causes of fatigue in adults with Neurofibromatosis type 1

**DOI:** 10.3389/fendo.2023.1119159

**Published:** 2024-03-15

**Authors:** Anna G. W. Rosenberg, Ké Mochèl, Lorena M. Hähner, Lara Ruules, Kirsten Davidse, Anja G. Bos-Roubos, Sarah A. van Dijk, M. Carola Zillikens, Walter Taal, Aart J. van der Lely, Laura C. G. de Graaff

**Affiliations:** ^1^ Department of Internal Medicine, Division of Endocrinology, Erasmus MC, University Medical Center Rotterdam, Rotterdam, Netherlands; ^2^ Center of Excellence for Neuropsychiatry, Vincent van Gogh, Venray, Netherlands; ^3^ Department of Neurology/Neuro-Oncology, Erasmus MC Cancer Institute, University Medical Center Rotterdam, Rotterdam, Netherlands; ^4^ ENCORE-Dutch Center of Reference for Neurodevelopmental Disorders, Rotterdam, Netherlands; ^5^ ERN BOND, European Reference Network for Rare Bone Diseases, Rotterdam, Netherlands; ^6^ ENDO-ERN, European Reference Network on Rare Endocrine Conditions, Rotterdam, Netherlands; ^7^ Academic Centre for Rare Bone Disorders, Erasmus MC, University Medical Center, Rotterdam, Netherlands

**Keywords:** Neurofibromatosis type 1 (NF1), fatigue, adult, internal medicine, endocrinology

## Abstract

**Context:**

Neurofibromatosis type 1 (NF1) is a complex system disorder, caused by alterations in RAS pathways. NF1 adults often suffer from chronic and severe fatigue, for which they are frequently referred to Internal Medicine/Endocrinology. Seeking medical help often leads to (invasive) diagnostic procedures. To prevent the personal and financial burden of this disabling fatigue, it is crucial to know the causes.

**Objective:**

To explore somatic causes and provide practical recommendations for the approach to fatigue in adults with NF1.

**Design:**

Cross-sectional. All adults with NF1 (N = 133) who visited our Endocrinology department underwent a systematic health screening, including a medical questionnaire, structured interview, complete physical examination, biochemical measurements and additional tests if indicated.

**Main outcome measure:**

Prevalence of endocrine and non-endocrine health problems between NF1 adults with and without fatigue.

**Results:**

In our cohort, 75% of NF1 adults experienced fatigue. The most frequent endocrine disorders were vitamin D deficiency (28%), obesity (18%) and hypothyroidism (8%). The most frequent non-endocrine internal disorder was high blood pressure (42%). None of the disorders differed significantly between adults with and without fatigue.

**Conclusions:**

Endocrine and non-endocrine disorders were equally present in our cohort of NF1 adults with and without fatigue. This suggests that the high prevalence of fatigue in NF1 adults is not explained by these somatic disorders. An alternative explanation for fatigue might be deficits in cognitive functioning and other neuropsychological processes in NF1. Based on our results and review of the literature, we provide a clinical algorithm for the approach to fatigue in NF1 adults, including somatic and psychological assessment.

## 1 Introduction

Neurofibromatosis type 1 (NF1) is a complex autosomal dominant neurocutaneous syndrome caused by mutations in the *NF1* gene on chromosome 17q11.2 (1). Since genetic testing can be labor intensive and expensive, the diagnosis is often clinically made when at least two characteristics of NF1, like café-au-lait macules and neurofibromas, are present (2). The estimated birth incidence of NF1 is approximately 1:2000 to 1:3500 (3). The clinical expression of NF1 is highly variable (4). People with NF1 have a predisposition to develop benign and malign tumors (5). The most frequent manifestations are café-au-lait macules (>99%), cutaneous or plexiform neurofibromas (>99%), Lisch nodules (>95%) and skinfold freckling (85%) (5). Furthermore, up to 60% of the people with NF1 have learning problems (5). Less frequent manifestations include scoliosis, optic pathway glioma, macrocephaly and short stature, among others (5).

Adults with NF1 frequently suffer from fatigue (6), which is often chronic and severe, interfering with daily functioning and causing absence from school or work (personal communications). Patients usually turn to their general practitioner for medical help and are frequently referred to Internal Medicine for analysis, where standard workup of chronic fatigue often includes, sometimes invasive, diagnostic procedures.

Fatigue can be defined as a subjective feeling of weakness with three components: (a) difficulty or inability to initiate activities (b), decreased capacity to continue activities and (c) difficulty with memory, concentration and emotional stability (“mental fatigue”) (7, 8).

In the general population, endocrine causes of fatigue include hypothyroidism, hyperthyroidism, hypogonadism, diabetes mellitus, adrenal insufficiency, obesity, vitamin D deficiency and electrolyte abnormalities (8). Non-endocrine somatic disorders that can possibly cause fatigue include renal and hepatic disease, anemia and cardiovascular diseases, among others (8). Besides, fatigue can also be related to deficits in neuropsychological functioning and mental health issues, medication and substance use (8). Apart from neurocognitive and psychosocial problems that are present in the general population (and might therefore be evenly present in patients with NF1), the clinical impression from pediatricians, internists and neurologists from our NF1 reference center is that neurocognitive and psychosocial difficulties are more pronounced in patients with NF1 than in the general population.

In NF1, possible causes of fatigue are yet largely unknown (9). Prevalences of diabetes mellitus, hepatic disease, anemia and electrolyte abnormalities have not been reported to our knowledge. Little is known about the pituitary function of adults with NF1. Pituitary hormone deficiencies have been reported in children with NF1 and were mostly related to the presence of optic pathway gliomas (10, 11). Furthermore, recent studies suggested that growth hormone excess might be more prevalent in children and adults with NF1 and that this is related to pituitary lesions (12, 13). One study among children with NF1 showed that thyroid function is normal (14), whereas another study showed that subclinical hypothyroidism was prevalent in NF1 (15).Vitamin D levels are generally lower in people with NF1 (16, 17) and lower levels of vitamin D have been associated with a higher number of neurofibromas (18, 19). However, this correlation is not always found (20) and the clinical relevance is also unknown as the true relation between vitamin D deficiency and fatigue is still subject to discussion. The mechanism behind the lower vitamin D levels has yet to be determined. Obesity can also (indirectly) cause fatigue, due to the increased risk of obstructive sleep apnea (OSA) in obese people or due to impairments in physical activity. Several studies have investigated body composition and body mass index (BMI) in NF1 (21–23) and reported that the prevalence of obesity is not higher in NF1 than in the general population. Fatigue can also be caused by pheochromocytomas, catecholamine-producing adrenal tumors, which are more frequent in NF1 than in the general population (24). Although most prevalent symptoms of pheochromocytoma include hypertension, palpitations and headache (25), it might also cause fatigue. Pheochromocytomas occur in 0.1 – 5.7% of people with NF1, but the incidence is as high as 20-56% when hypertension is present (compared to 0.1% in hypertensive individuals without NF1) (24, 26). Lastly, cardiovascular and renal problems associated with NF1 (27, 28) may also cause fatigue.

Overall, data on somatic causes of fatigue in adults with NF1 are very scarce, while the high prevalence of fatigue in adults with NF1 makes fatigue an important topic. To prevent the personal and financial burden of the disabling fatigue and the associated diagnostic trajectory it is crucial to know the etiology. In this study, we have explored endocrine and non-endocrine causes and provide practical recommendations for the approach to fatigue in adults with NF1.

## 2 Materials and methods

Approval for this cross-sectional study was waived by the local medical ethics committee of the Erasmus University Medical Center Rotterdam. We retrospectively collected data of all adults with NF1 who visited the Endocrinology department at the Erasmus University Medical Center, Rotterdam, the Netherlands, between April 2016 and December 2021.

### 2.1 Systematic health screening

All adults underwent a systematic health screening during their first visit to our outpatient clinic. The screening consisted of a medical questionnaire, structured interview, complete physical examination, review of the medical records, biochemical analysis and additional tests if indicated (such as dual energy X-ray absorptiometry).

#### 2.1.1 Medical questionnaire

The questionnaire included items on medical history (past operations and co-morbidities), medication and use of supplements, family history, physical complaints, lifestyle (intoxications, physical activity, intake of dairy products) and social aspects (work, school, relationship, living situation). Frequency and severity of physical complaints were rated on a 5-point Likert-scale (1 = rarely or never, 2 = not often and/or not severe, 3 = quite often and/or quite severe, 4 = often and/or severe, 5 = very often and/or very severe). A score of 3 or higher was considered clinically relevant and further explored during the visit to the outpatient clinic.

#### 2.1.2 Biochemical analysis

During the first visit, venous blood samples were taken for general medical screening, including evaluation of thyroid function (free thyroxine (fT4), thyroid stimulating hormone (TSH)), gonadal function (follicle stimulating hormone (FSH), luteinizing hormone (LH), estradiol or testosterone, sex hormone-binding globulin (SHBG)), glucose metabolism (non-fasting glucose, glycated hemoglobin (HbA1c)), fat metabolism (low-density lipoprotein cholesterol (LDL)), liver function (alanine aminotransferase (ALAT), aspartate transaminase (ASAT), alkaline phosphatase (ALP), gamma-glutamyl transferase (GGT), lactate dehydrogenase (LDH), total bilirubin), kidney function (creatinine, albumin, urea, estimated glomerular filtration rate (eGFR)), hematopoietic system (hemoglobin (Hb), mean corpuscular volume (MCV), red cell distribution width (RDW)), electrolytes (sodium (Na), potassium (K), calcium (Ca)), 25-OH-Vitamin D status, insulin-like growth factor 1 (IGF-1), prolactin and cortisol. Metanephrines and normetanephrines were measured when symptoms of pheochromocytoma (hypertension and/or palpitations) were present. Calcium levels were corrected for albumin when they were below or above the reference range. Parathyroid hormone (PTH) levels were measured when albumin corrected calcium levels were elevated or decreased. Reference values are presented in the **Supplementary Data, Table S1**. Only results of blood samples that were taken maximally one year before or after the first visit to our outpatient clinic were collected for this study.

### 2.2 Fatigue

Fatigue was defined as a subjective feeling of (extreme) tiredness. When patients scored ≥ 3 on ‘feeling tired’ in the medical questionnaire, the perceived fatigue was discussed during their first visit to the outpatient clinic. Patients were classified as having fatigue when the feelings of tiredness were confirmed during the structured interview. Patients who scored <2 on ‘feeling tired’ in the medical questionnaire were classified as not having fatigue, unless they explicitly mentioned feelings of tiredness during the structured interview. In that case, they were classified as having fatigue. When the questionnaire or the question about fatigue was not filled out, patients were classified as having fatigue when feelings of tiredness were explicitly mentioned during their first visit to the outpatient clinic. All other patients were classified as not having fatigue.

### 2.3 Health problems

We explored the association of fatigue with the following physical health problems: thyroid disease, hypogonadism, pheochromocytoma, low eGFR, liver enzyme disturbances, cardiovascular disorders, electrolyte disorders, vitamin D deficiency, anemia, obesity, underweight and the presence of cutaneous neurofibromas.

Thyroid disease was subdivided into four groups: overt hypothyroidism, subclinical hypothyroidism, overt hyperthyroidism and subclinical hyperthyroidism. When a patient was already known with thyroid disease, the patient was included in the relevant group. When a patient was not known with thyroid disease, biochemical measurements (fT4 and TSH) were evaluated. The reference values for the laboratory measurements are given in the **Supplementary Data, Table S1**. Only when laboratory measurements were repeatedly below or above the reference range, thyroid disease was diagnosed. Overt hypothyroidism was diagnosed when fT4 was low with increased TSH. Subclinical hypothyroidism was diagnosed when fT4 was normal with increased TSH. Overt hyperthyroidism was diagnosed when fT4 was high with decreased TSH. Subclinical hyperthyroidism was diagnosed when fT4 was normal with decreased TSH.

Classification of hypogonadism was also based on clinical features (absence of/irregular menstrual cycle in females, erectile dysfunction and little facial and body hair in males) combined with biochemical measurements (low testosterone for men; low estrogen for women) and previous prescription of testosterone or estrogen supplementation. Hypogonadism was only diagnosed when the patient was already known with hypogonadism or when biochemical measurements (testosterone in men, estrogen in women) were repeatedly below the reference value (**Supplementary Data, Table S1**). When hypogonadism was present, biochemical measurements were used to distinguish primary hypogonadism (low testosterone or estrogen combined with high FSH and/or LH) and secondary hypogonadism (low testosterone or estrogen combined with low FSH and/or LH). Patients who used oral contraceptives or had reached menopausal age were analyzed separately.

Diabetes was considered present when patients were already known with diabetes mellitus. When patients were not known with diabetes mellitus, biochemical measurements were evaluated. When non-fasting glucose levels were repeatedly above the upper limit (**Supplementary Data, Table S1**), fasting glucose levels were measured. When these were also elevated (≥7.0 mmol/L), diabetes mellitus was considered present.

Pheochromocytoma was diagnosed when plasma (nor-)metanephrine levels were elevated and (nuclear) imaging showed a pheochromocytoma. The plasma (nor-)metanephrine levels were used as a first screening. Only when these levels were repeatedly elevated, (nuclear) imaging was performed for the diagnosis of pheochromocytoma.

Kidney function was assessed with biochemical measurements. When eGFR was repeatedly below the reference value (**Supplementary Data, Table S1**), the patient was considered having ‘low eGFR’.

For liver enzyme disturbances, biochemical measurements were used to distinguish three categories: (a) GGT and ALP elevation, (b) high total bilirubin (often accompanied with high GGT and ALP), and (c) other liver enzyme disorders. Isolated GGT elevation and isolated ALP elevation were separately analyzed.

Classification of cardiovascular disorders was based on previous clinical diagnosis of cardiovascular disease or use of cardiac medication. An elevated blood pressure (systolic blood pressure ≥ 140 mmHg and/or diastolic blood pressure ≥ 90 mmHg) was not considered a ‘cardiovascular disorder’, but was analyzed separately.

For electrolyte disorders, we screened for elevated or decreased levels of calcium, sodium and potassium. When calcium levels were abnormal, PTH levels were measured to rule out hypoparathyroidism (low calcium ánd low or normal PTH) or hyperparathyroidism (high calcium ánd high or normal PTH).

Patients with low (< 50 nmol/L) vitamin D levels (without supplementation) were classified as having vitamin D deficiency. Since vitamin D supplements are sometimes prescribed despite (low-)normal vitamin D levels, for example in case of osteoporosis, adults who used vitamin D supplements were analyzed separately.

Anemia was categorized into three categories based on Hb and MCV measurements: microcytic anemia (low Hb and MCV <80 fL), normocytic anemia (low Hb and MCV of 80 – 100 fL) and macrocytic anemia (low Hb and MCV >100 fL).

Obesity was defined as BMI ≥30 kg/m^2^, overweight as BMI between 25 and 30 kg/m^2^, and underweight as BMI <18.5 kg/m^2^.

### 2.4 Literature search

Literature was searched in the databases ‘Embase’, ‘Medline ALL’, ‘Web of Science Core Collection’, ‘Cochrane Central Register of Controlled Trials’ and ‘PsycINFO’. The search was last updated on October 25th, 2021. We included all original research articles meeting the following criteria: (1) people with NF1 and (2) fatigue as outcome, regardless of the genetic subtype, comorbidities, or age. Exclusion criteria were: (1) non-original research articles, (2) articles without full-text availability and (3) questionnaire validation studies. There were no restrictions on language or period. All articles were screened by two independent researchers (AR, KM), first by title and abstract, and then, if relevant, by full-text. In case of disagreement, a third researcher (LdG) was consulted. The full search strategy is available upon request.

### 2.5 Data analysis

Data were analyzed with IBM Statistical Package for the Social Sciences Statistics for Windows version 28.0 and R version 3.6.0. Continuous data are presented as mean ± standard deviation (SD) for normally distributed data and as median [interquartile range (IQR)] for non-normally distributed data. Categorical data are presented as count and percentage (%). Wilcoxon rank sum test (non-normally distributed data) or unpaired t-test (normally distributed data) were used to detect differences in continuous data between people with and without fatigue. Logistic regression analyses were performed to detect differences in categorical data between people with and without fatigue. P-values <.05 were considered significant.

## 3 Results

One-hundred-thirty-three adults with NF1 (39 males, 94 females) visited the outpatient clinic, of whom 100 (75%) experienced fatigue. The medical questionnaire was filled out by 110 (83%) adults. The patient characteristics are described in **Table 1**. Median age was 33 years (IQR 25 – 49 years) and median BMI was 24.9 kg/m^2^ (IQR 21.9 – 28.7 kg/m^2^). Most adults followed secondary vocational education (N = 51; 38%). More adults with fatigue (N = 20; 20%) followed special education compared to adults without fatigue (N = 2; 6%; P = 0.07).

**Table 1:** Characteristics of adults with Neurofibromatosis type 1.

	All (N = 133)	Fatigue (N = 100)	No fatigue (N = 33)
Gender			
Male, N (%)	39 (29%)	29 (29%)	10 (30%)
Female, N (%)	94 (71%)	71 (71%)	23 (70%)
Age (years)			
Median	33.0	33.0	33.0
IQR	25.0 – 49.0	25.0 – 49.0	28.0 – 52.0
BMI (kg/m^2^)			
Median	24.9	24.8	25.6
IQR	21.9 – 28.7	21.7 – 27.9	24.1 – 30.0
Systolic blood pressure (mmHg)^a^			
Mean ± SD	134.2 ± 16.1	134.5 ± 16.6	133.5 ± 14.7
Diastolic blood pressure (mmHg)^a^			
Mean ± SD	80.0 ± 11.9	78.9 ± 11.5	83.2 ± 12.7
Scholar level			
Primary school, N (%)	3 (2%)	1 (1%)	2 (6%)
Special education, N (%)	22 (17%)	20 (20%)	2 (6%)
Prevocational secondary education, N (%)	12 (9%)	9 (9%)	3 (9%)
Secondary vocational education, N (%)	51 (38%)	39 (39%)	12 (36%)
Senior general secondary education/pre-university education, N (%)	3 (2%)	2 (2%)	1 (3%)
Higher vocational education/research university, N (%)	26 (20%)	18 (18%)	8 (24%)
Unknown, N (%)	16 (12%)	11 (11%)	5 (15%)
Exercise (daily)			
<30 minutes, N (%)	30 (23%)	25 (25%)	5 (15%)
30 – 60 minutes, N (%)	45 (34%)	34 (34%)	11 (33%)
>60 minutes, N (%)	35 (26%)	25 (25%)	10 (30%)
Unknown, N (%)	23 (17%)	16 (16%)	7 (21%)
Dairy intake (daily)			
<2 units, N (%)	54 (41%)	45 (45%)	9 (27%)
2 – 3 units, N (%)	45 (34%)	35 (35%)	10 (30%)
>3 units, N (%)	11 (8%)	7 (7%)	4 (12%)
Unknown, N (%)	23 (17%)	13 (13%)	10 (30%)
Alcohol use			
Yes, N (%)	84 (63%)	59 (59%)	25 (76%)
No, N (%)	44 (33%)	38 (38%)	6 (18%)
Unknown, N (%)	5 (4%)	3 (3%)	2 (6%)
Smoking			
Yes, N (%)	22 (17%)	17 (17%)	5 (15%)
No, N (%)	107 (80%)	81 (81%)	26 (79%)
Unknown, N (%)	4 (3%)	2 (2%)	2 (6%)
Drug use			
Yes, N (%)	10 (8%)	8 (8%)	2 (6%)
No, N (%)	116 (87%)	88 (88%)	28 (85%)
Unknown, N (%)	7 (5%)	4 (4%)	3 (9%)

BMI, body mass index.

None of the variables differed significantly between adults with and without fatigue. ^a^Data on blood pressure were missing in 7 adults of whom all experienced fatigue.

We assessed whether patients met the generally prescribed lifestyle recommendations and found that most patients exercised for 30-60 minutes per day (N = 45; 34%) and that 41% had a daily dairy intake of <2 units per day. Furthermore, 17% (N = 22) of the adults smoked, 8% (N = 10) used drugs and 63% (N = 84) used alcohol. There were no significant differences in lifestyle between adults with and without fatigue.

The prevalence of endocrine and non-endocrine disorders related to fatigue and an overview of laboratory measurements are shown in **Table 2**, **Supplementary Data, Table S4**. The most frequent endocrine-related problems were hypothyroidism (N = 11; 8%; 95% CI 5% - 15%), obesity (N = 24; 18%, 95% CI 12% - 25%) and vitamin D deficiency (N = 10; 28%; 95% CI 16% - 44%). Up to 70% (N = 86; 95% CI 62% - 78%) of the adults received vitamin D supplementation. Vitamin D deficiency was not related to the presence of neurofibromas in our cohort (see also **Supplementary Data, Table S3**). Although an elevated blood pressure was seen in 42% (N = 53; 95% CI 34% - 51%), pheochromocytoma was only present in 2 adults. In 7 adults with elevated blood pressure, (nor)metanephrine levels could not be measured. However, they had no other symptoms associated with pheochromocytoma. None of the disorders differed significantly between adults with and without fatigue.

**Table 2:** Prevalence of endocrine and non-endocrine disorders in relation to fatigue in adults with Neurofibromatosis type I.

	All (N = 133)			Fatigue (N = 100)		No fatigue (N = 33)		P-value
	N (%)	Missing	95% CI	N (%)	Missing	N (%)	Missing	
Endocrine disorders								
Thyroid disorders								
Hypothyroidism	11 (8%)	3	5% - 15%	7 (7%)	1	4 (13%)	2	0.28
Subclinical hypothyroidism	3 (2%)	3	1% - 7%	1 (1%)	1	2 (6%)	2	0.11
Hyperthyroidism	5 (4%)	3	2% - 9%	4 (4%)	1	1 (3%)	2	0.92
Subclinical hyperthyroidism	1 (1%)	3	0% - 4%	1 (1%)	1	0 (0%)	2	0.99
Parathyroid disorders								
Hypoparathyroidism	0 (0%)	17	0% - 3%	0 (0%)	12	0 (0%)	5	N/A
Hyperparathyroidism	1 (1%)	17	0% - 5%	0 (0%)	12	1 (4%)	5	0.99
Hypogonadism								
Central hypogonadism^a^	1 (1%)	4	0% - 4%	0 (0%)	4	1 (5%)	0	0.99
Primary hypogonadism^a^	0 (0%)	4	0% - 3%	0 (0%)	4	0 (0%)	0	N/A
Subclinical hypogonadism^a^	3 (4%)	4	1% - 7%	3 (5%)	4	0 (0%)	0	0.99
Use of oral contraceptives^b^	30 (33%)	4	24% - 43%	23 (34%)	3	7 (30%)	0	0.56
Menopausal age^b^	23 (25%)	3	17% - 35%	16 (24%)	3	7 (30%)	0	0.97
Diabetes mellitus type 2	2 (2%)	2	0% - 5%	2 (2%)	2	0 (0%)	0	0.99
Obesity	24 (18%)	0	12% - 25%	16 (16%)	0	8 (24%)	0	0.29
Underweight	3 (2%)	0	1% - 6%	3 (3%)	0	0 (0%)	0	0.99
Pheochromocytoma^c^	2 (4%)	1	0% - 5%	2 (6%)	1	0 (0%)	0	0.38
Vitamin D								
Vitamin D deficiency^d^	10 (28%)	11	16% - 44%	8 (32%)	9	2 (18%)	2	0.40
Vitamin D supplementation	86 (70%)	11	62% - 78%	66 (73%)	9	20 (65%)	2	0.25
Electrolyte disorders								
Hypo- or hyperkalemia	0 (0%)	8	0% - 3%	0 (0%)	4	0 (0%)	4	N/A
Hyponatremia	1 (1%)	7	0% - 4%	0 (0%)	4	1 (3%)	3	0.99
Hypernatremia	4 (3%)	7	1% - 8%	4 (4%)	4	0 (0%)	3	0.99
Hypocalcemia	2 (2%)	17	0% - 6%	0 (0%)	12	2 (7%)	5	0.99
Hypercalcemia	1 (1%)	17	0% - 5%	0 (0%)	12	1 (4%)	5	0.99
Non-endocrine disorders								
Low eGFR	11 (9%)	6	5% - 15%	8 (8%)	4	3 (10%)	2	0.82
Liver enzyme disturbances								
High total bilirubin	14 (11%)	8	7% - 18%	13 (14%)	5	1 (3%)	3	0.14
GGT ánd ALP elevation	7 (6%)	8	3% - 11%	5 (5%)	5	2 (7%)	3	0.92
Other liver enzyme dysfunction	8 (6%)	8	3% - 12%	7 (7%)	5	1 (3%)	3	0.39
Isolated GGT elevation	9 (7%)	8	4% - 13%	7 (7%)	5	2 (7%)	3	0.77
Isolated ALP elevation	5 (4%)	8	2% - 9%	3 (3%)	5	2 (7%)	3	0.53
Cardiac disorders	15 (11%)	0	7% - 18%	12 (12%)	0	3 (9%)	0	0.65
Elevated blood pressure	53 (42%)	7	34% - 51%	37 (40%)	7	16 (48%)	0	0.39
Anemia								
Microcytic	4 (3%)	4	1% - 8%	2 (2%)	1	2 (7%)	3	0.21
Normocytic	10 (8%)	4	4% - 14%	7 (7%)	1	3 (10%)	3	0.55
Macrocytic	0 (0%	4	0% - 3%	0 (0%)	1	0 (0%)	3	N/A
Cutaneous neurofibromas	103 (88%)	16	81% - 93%	81 (90%)	10	22 (81%)	6	0.24

ALP, alkaline phosphatase; eGFR, estimated glomerular filtration rate; GGT, gamma-glutamyl transferase.

^a^Adults who used oral contraceptives or had reached menopausal age were excluded from the analyses. ^b^Males were excluded from the analyses. ^c^Metaneprhine and normetanephrine levels were available in 46 adults, of whom 34 with fatigue and 12 without fatigue. Nuclear imaging was missing in 1 adult (with fatigue). ^d^Adults who used vitamin D supplementation were excluded from the analysis.

Neurological data (speech, cranial nerves, motor function, sensory function, coordination, gait, reflexes and pain) were present in a subset of our cohort (N = 53). The results are shown in the **Supplementary Data, Table S5**. There were no significant differences between adults with and without fatigue.

Our literature search yielded 192 articles of which 140 remained after deduplication. Eleven articles were identified as potentially relevant. After full-text screening, four articles were included. One additional article was found through cross references. The characteristics and findings of the included articles are described in **Table 3**.

**Table 3:** Study characteristics and results of previous literature about fatigue in people with Neurofibromatosis type 1.

Author	Study design	Number of participants (N)	Age in years (mean ± SD)	Gender (N)	Results	Remarks
Vassallo et al. (2020) (9)	Case-control	75 NF1, 16 control	Range: 2 - 18	35 M, 40 F (NF1) 9 M, 7 F (control)	Aggregated fatigue score was higher among children with NF compared to controls (*P* <.001 for child report, *P* = .001 for parent report). Scores for the individuals subdomains “cognitive”, “physical” and “sleep/rest” were also significantly higher among NF1 children. The prevalence of perceived severe fatigue in NF1 was 69% (parent report) and 34% (child report).	Low response rate of 24% may indicate selection bias.
Fjermestad et al. (2019) (29)	Cross-sectional	142	50.3 ± 12.0	54 M, 88 F	74% of participants experienced work as physically exhausting compared to 44% of the control group (Nord-Trøndelag Health Study; *P* <.005).	Only physical fatigue related to physically demanding work.
Lai et al. (2019) (30)	Cross-sectional	140	12.5 ± 2.7	90 M, 50 F	On average, children with NF1-related plexiform neurofibromas did not experience more fatigue than the general population (using PROMIS).	Only children with NF1-related plexiform neurofibromas were included.
Talaei-Khoei et al. (2017) (31)	Cross-sectional	43	44.1 ± 15.3	N/A	On average, there was no difference in the fatigue score between adults with NF1 and the general population (using PROMIS).	N/A
Kodra et al. (2009) (32)	Cross-sectional	129	37.7 ± 12.2	51 M, 78 F	Approximately 18% of the people with NF1 reported feeling tired often or all the time.	Exact numbers were not given.

F, female; M, male; NF1, Neurofibromatosis type 1; PROMIS, patient reported outcomes measurement information system.

**Figure 1:**
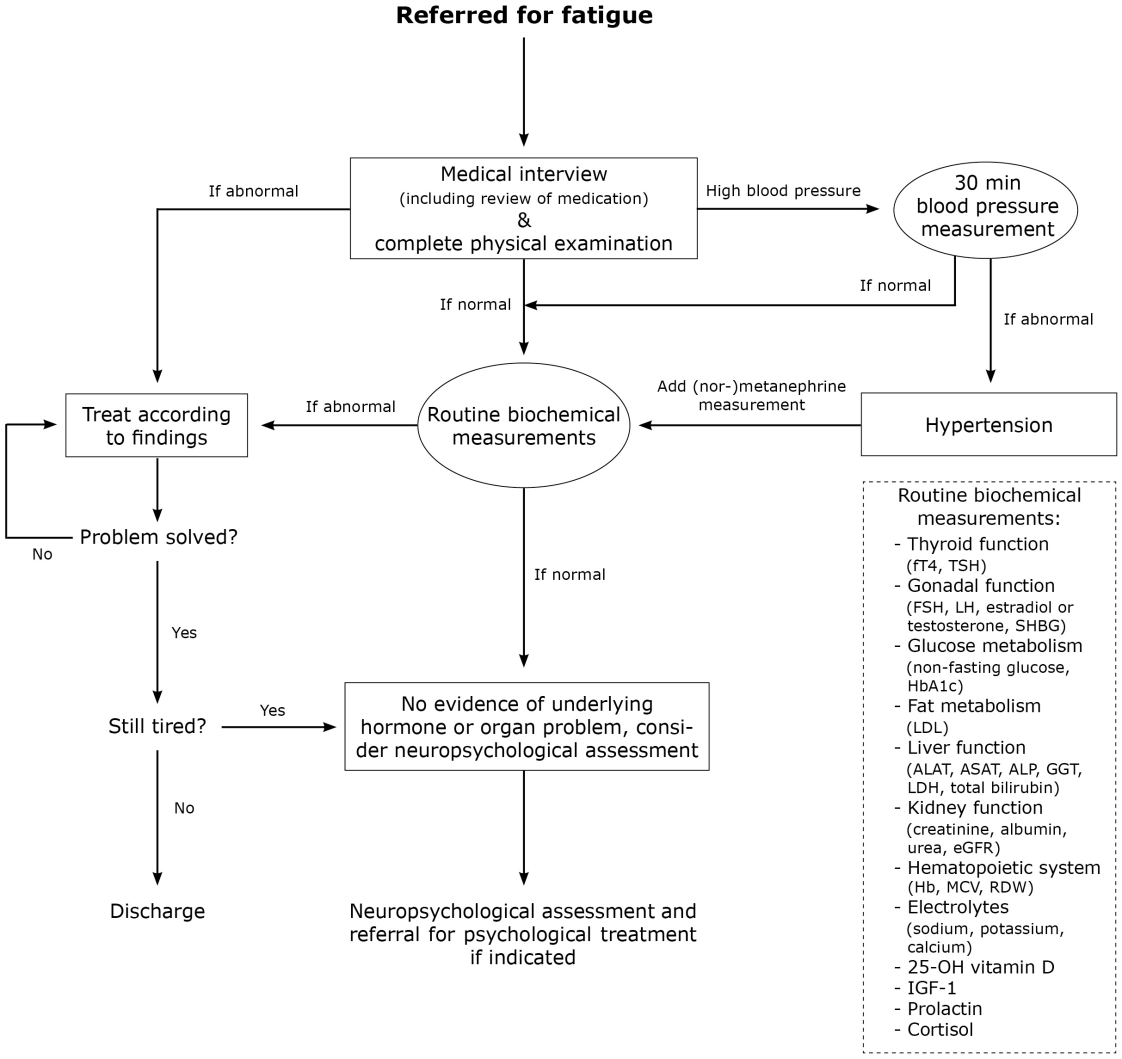
Algorithm for the approach to fatigue in adults with Neurofibromatosis type 1. ALAT, alanine aminotransferase; ALP, alkaline phosphatase; ASAT, aspartate aminotransferase; eGFR, estimated glomerular filtration rate; FSH, follicle stimulating hormone; fT4, free thyroxine 4; GGT, gamma-glutamyl transferase; Hb, hemoglobin; HbA1c, glycated hemoglobin; IGF-1, insulin-like growth factor 1; LDH, lactate dehydrogenase; LDL, low-density lipoprotein; LH, luteinizing hormone; MCV, mean corpuscular volume; RDW, red cell distribution width; SHBG, sex hormone-binding globulin; TSH, thyroid stimulating hormone.

Based on our findings and the previous literature, we developed an algorithm for the approach to fatigue in adults with NF1 (**Figure 1**).

## 4 Discussion

In our cohort, NF1 adults often suffer from chronic and severe fatigue, possibly leading to reduced quality of life and high healthcare consumption in this population. Understanding the etiology is crucial to prevent the personal and financial burden of this disabling fatigue. We studied endocrine and non-endocrine causes of fatigue in 133 adults with NF1. In our cohort, none of the endocrine and non-endocrine disorders differed significantly between adults with and without fatigue. Supported by our literature overview, we conclude that fatigue is a common, yet somatically unexplained, complaint. We hypothesize that psychological aspects (cognitive and other neuropsychological deficits) might play an important role in the etiology of fatigue in adults with NF1.

The prevalence of hypothyroidism was slightly higher in our cohort (8%; 95% CI 5% - 15%) compared to the general Dutch population (approximately 3%) (33). However, no difference was seen between NF1 adults with and without fatigue. The most frequent endocrine-related disorder in our cohort was vitamin D deficiency (28%; 95% CI 16% - 44%). However, the true prevalence might be higher as many adults (N = 86; 70%) used vitamin D supplements. Several studies found that the prevalence of vitamin D deficiency is higher in people with NF1 compared to the general population (16, 17, 34). Since the prevalence of vitamin D deficiency depends on the season it was not possible to compare the prevalence of vitamin D deficiency in our cohort with the general population, as we had no data on the season. Some studies found that lower vitamin D levels in NF1 are related to a higher number of dermal neurofibromas (18, 19, 35). This suggests that a functional ‘loss’ of vitamin D might contribute to the development of neurofibromas. However, another study did not find a correlation between the number of dermal neurofibromas and vitamin D levels (20). Therefore, more research is needed on this topic. Apart from skin factors, lifestyle could contribute to the onset of vitamin D deficiency. For example, lack of intake of vitamin D in the diet or lack of exposure to sunlight could lead to inadequate vitamin D levels (36). Especially people with NF1 may have less exposure to sunlight due to cutaneous manifestations of the disease, including neurofibromas. Presence of neurofibromas and other dermal manifestations of NF1 may lead to more skin covering, possibly in combination with decreased outdoor activities due to lower self-confidence, lower self-esteem, anxiety, and depression. However, according to a German study that compared vitamin D levels between people with and without NF1, it is unlikely that lack of sunlight exposure *alone* explains the lower vitamin D levels (37). Therefore, more research on vitamin D levels in adults with NF1 is needed.

Also medication can cause or aggravate fatigue. Especially analgesics, cholesterol synthesis inhibitors, selective beta blockers, antidepressants, proton-pump inhibitors, antihistamines and benzodiazepines have been associated with fatigue in general (38). Therefore, thorough review of medication should be part of routine clinical care for NF1 adults with fatigue.

Beside the endocrine and non-endocrine somatic causes for fatigue that we assessed in this study, there are other somatic factors that could be involved in the etiology of fatigue. Recent studies showed that people with NF1 may have muscle dysfunction due to intramyocellular lipid storage, which could contribute to fatigue (39, 40). One study also suggested that people with NF1 might be more prone to develop premature sarcopenia, possibly contributing to fatigue (21). Furthermore, it has been suggested that people with NF1 might have a predisposition to autoimmunity leading to autoimmune disorders like multiple sclerosis or systematic lupus erythematosus, which could also lead to fatigue (41–44). Besides, one study reported a high prevalence of obstructive sleep apnea in people with NF1, possibly leading to feelings of tiredness (45). Lastly, NF1 has been associated with lower vitamin B12 levels, which could contribute to fatigue (46).

In this study, we focused on endocrine and other somatic causes for fatigue. However, fatigue can also be the result of (neuro-)psychological alterations. Some adults with NF1 have a distinct neuropsychological profile with lower visual-spatial skills and auditory long-term memory, and impaired executive functioning such as planning, working memory, time management and organization (47). In addition, a recent population-based study by Kenborg et al. (48) showed that people with NF1 have a higher risk of developing a psychiatric disorder. Especially attention deficit hyperactivity disorder and depression have been associated with NF1 (48, 49). Both depression and lowered executive functions are related to a lower quality of life in people with NF1 (49, 50). We hypothesize that (neuro-)psychological alterations may contribute to the high prevalence of fatigue in adults with NF1. Therefore, psychological counseling should be considered during the diagnostic work-up of fatigue in adults with NF1. Neuropsychological assessment and, if needed, adaptation of daily tasks and activities might help reduce fatigue. Future research on the relation between neuropsychological alterations and fatigue is needed to further unravel the etiology of fatigue in adults with NF1.

This is the first study to focus on endocrine and non-endocrine causes of fatigue in adults with NF1. Strengths of our study are the relatively large sample size (considering that NF1 is a rare disorder) and the focus on adults only. Our biggest strength, however, is that all patients underwent a systematic health screening by the same physician and nurse practitioner. Because of this systematic approach, there were no differences in follow-up, diagnostics and treatment between people with and without fatigue, which reduced bias of the treating physician/nurse practitioner. However, some limitations remain. Most importantly, many patients were referred to our center for analysis of their fatigue or high blood pressure. Therefore, the percentages of adults with fatigue (75%) and high blood pressure (42%) are high and not representative for the general population of adults with NF1. Given the fact that many adults visited our center for analysis of their fatigue, we did not aim to assess the prevalence of fatigue in adults with NF1, but only to analyze the possible somatic causes for fatigue. Furthermore, the high prevalence of elevated blood pressure could also be partly due to a ‘white coat effect’. Besides, we had no data on the number of cutaneous neurofibromas, the presence of plexiform neurofibromas, bone mineral density, vitamin B12 levels and malignancies. Although we had a relatively large sample size, absolute numbers were generally low and small differences may still exist. Moreover, our results are a representation of a group of adults with NF1, which means that individual differences still exist and that no individual conclusions can be drawn from this study. Therefore, our results should be interpreted with caution.

In our study, we show that there are no differences in somatic (endocrine and non-endocrine) disorders between NF1 adults with and without fatigue who attended our outpatient clinic. We hypothesize that neuropsychological factors might contribute to fatigue in adults with NF1. Therefore, we recommend to perform clinical neuropsychological assessment when a first somatic screening shows no evidence of underlying hormone or organ problems. We recommend to follow the practical algorithm for the approach to fatigue in adults with NF1 (**Figure 1**) and to refrain from extensive diagnostics to find a somatic cause, considering the lack of added value and high patient burden.

### 4.1 Conclusion

Based on our results, fatigue is a common, but somatically unexplained, complaint in adults with NF1. In our cohort, the assessed endocrine and non-endocrine disorders are equally present in NF1 adults with and without fatigue. This suggests that the high prevalence of fatigue in adults with NF1 is not explained by these somatic disorders. We hypothesize that neuropsychological aspects may plan in important role in the etiology of somatically unexplained fatigue in adults with NF1. Based on our results and review of the literature, we provide a clinical algorithm for the approach to fatigue in adults with NF1, including somatic and psychological assessment.

## Data availability statement

All datasets generated for this study are available upon reasonable request from the corresponding author. Requests to access the datasets should be directed to L.C.G. de Graaff.

## Ethics statement

The studies involving humans were approved by the medical ethics committee of the Erasmus University Medical Center Rotterdam, 3035 GD Rotterdam, the Netherlands. The studies were conducted in accordance with the local legislation and institutional requirements. The ethics committee/institutional review board waived the requirement of written informed consent for participation from the participants or the participants’ legal guardians/next of kin because patients were not subject to procedures and were not required to follow rules of behavior. Patients were aware of the study and were given the opportunity to refuse participation.

## Author contributions

AR analyzed the data and wrote the first draft of the manuscript. AR, KM, LH, LR, and LD collected the data. AR and KM selected and reviewed the literature. LD was responsible for the conception of the study. AR and LD were responsible for the design of the study. All authors were involved in data interpretation, revision of the manuscript, and final approval of the manuscript.
