# Supplementary material for: Endocrine and non-endocrine causes of fatigue in adults with Neurofibromatosis type 1
Source: Front Endocrinol (Lausanne). 2024 Mar 15;14:1119159. doi: 10.3389/fendo.2023.1119159 (PMC10978575; doi:10.3389/fendo.2023.1119159)
Supplement: Supplementary file 1 [file Presentation_1.pdf]

Supplementary data

Title: Endocrine and non-endocrine causes of fatigue in adults with Neurofibromatosis type I

Authors: Anna G.W. Rosenberg, Ké Mochèl, Lorena M. Hähner, Lara Ruules, Kirsten Davidse, Anja G. Bos-Roubos, Sarah A. van Dijk, M. Carola Zillikens, Walter Taal, Aart J. van der Lely, Laura C.G. de Graaff

Corresponding author: Laura C.G. de Graaff, MD, PhD; Department of Internal Medicine-Endocrinology, Erasmus University Medical Center. Dr. Molewaterplein 40, 3015 GD Rotterdam, the Netherlands. E-mail: l.degraaff@erasmusmc.nl

Table of contents

Table S1. Reference values for laboratory measurements

Table S2. Types of cardiac disorders

Table S3. Vitamin D deficiency and presence of neurofibromas

Table S4. Laboratory measurements of 133 adults with Neurofibromatosis type 1

Table S5. Neurological examination in relation to fatigue in adults with Neurofibromatosis type 1

References

Table S1. Reference values for laboratory measurements

**Laboratory measurement**	**Reference value**
Thyroid function	
TSH	0.4-4.3 mU/L before 1 February 2019 and 0.56-4.27 mU/L after that date
fT4	11-25 pmol/L before 12 April 2019 and 13.5-24.3 pmol/L after that date
PTH	0.68 – 4.40 pmol/L
Gonadal function	
FSH	2.0 – 7.0 IU/L before 1 February 2019 and 0.8 – 5.1 IU/L after that date
LH	1.0 – 5.5 IU/L before 1 February 2019 and 1.5 – 8.0 after that date
Estradiol	55 – 1285 pmol/L for 14 – 50 yr. old women and <55 pmol/L (women) >50 yr. old before 22 October 2020, and 100 - 1200 pmol/L for 14 – 50 yr. old women and <300 pmol/L (women) >50 yr. old after that date
Testosterone	10.0 – 30.0 nmol/L
SHBG	10 – 70 nmol/L (men) and 20 – 120 nmol/L (women)
Glucose metabolism	
Non-fasting glucose	<7.8 mmol/L (normal), 7.8 – 11.0 mmol/L (impaired glucose tolerance), ≥ 11.1 mmol/L (diabetes mellitus)
HbA1c	26 – 42 mmol/mol
IGF-1	18 – 19 yr. old: 19.2 – 66.0 nmol/L (men) and 15.7 – 64.9 nmol/L (women)<br>19 – 20 yr. old: 18.3 – 61.8 (men) and 14.8 = 50.2 nmol/L (women)<br>20 – 22 yr. old: 16.3 – 57.5 nmol/L (men) and 13.1 – 55.6 nmol/L (women)<br>22 – 25 yr. old: 14.1 – 49.1 nmol/L (men) and 11.4 – 47.4 nmol/L (women)<br>25 – 30 yr. old: 12.5 – 41.3 nmol/L (men) and 10.2 – 39.6 nmol/L (women)<br>30 – 35 yr. old: 11.6 – 34.8 nmol/L (men) and 9.6 – 33.9 nmol/L (women)<br>35 – 40 yr. old: 10.8 – 31.9 nmol/L (men) and 8.9 – 31.5 nmol/L (women)<br>40 – 45 yr. old: 9.7 – 30.0 nmol/L (men) and 8.0 – 28.7 nmol/L (women)<br>45 – 55 yr. old: 7.9 – 28.1 nmol/L (men) and 6.8 – 26.5 nmol/L (women)<br>55 – 65 yr. old: 6.4 – 26.5 nmol/L (men) and 5.4 – 24.3 nmol/L (women)<br>65 – 70 yr. old: 6.0 – 25.9 nmol/L (men) and 5.1 – 22.0 nmol/L (women)<br>70 – 75 yr. old: 5.3 – 24.7 nmol/L (men) and 4.8 – 22.1 nmol/L (women)<br>75 – 90 yr. old: 4.3 – 23.4 nmol/L (men) and 4.3 – 23.8 nmol/L (women)
Prolactin	0.1 – 0.98 U/L (premenopausal women), 0.06 – 0.93 U/L (postmenopausal women) and <0.36 U/L (men) before 1 February 2019, and 0.2 – 0.7 U/L (premenopausal women), 0.1 – 0.4 U/L (postmenopausal women) and 0.1 – 0.5 U/L (men) after that date
Cortisol	200 – 700 nmol/L
Pheochromocytoma	
Metanephrine	0.07 – 0.33 nmol/L
Normetanephrine	0.23 – 1.07 nmol/L
25-OH-vitamin D	50 – 120 nmol/L (normal), 30 – 50 nmol/L (mild deficiency), <30 nmol/L (severe deficiency)
Fat metabolism	
LDL cholesterol	18 – 19 yr. old: 1.3 – 3.6 mmol/L (men) and 1.4 – 3.9 mmol/L (women)<br>20 – 24 yr. old: 1.4 – 3.9 mmol/l (men) and women<br>25 – 29 yr. old: 1.6 – 4.3 mmol/L (men) and 1.4 – 3.9 mmol/L (women)<br>30 – 34 yr. old: 1.8 – 4.6 mmol/L (men) and 1.4 – 4.0 mmol/L (women)<br>35 – 39 yr. old: 1.9 – 4.8 mmol/L (men) and 1.5 – 4.1 mmol/L (women)<br>40 – 44 yr. old: 2.0 – 5.0 mmol/L (men) and 1.6 – 4.3 mmol/L (women)<br>45 – 49 yr. old: 2.1 – 5.1 mmol/L (men) and 1.8 – 4.6 mmol/L (women)

	50 – 54 yr. old: 2.1 – 5.1 mmol/L (men) and 2.0 – 5.0 mmol/L (women)<br>55 – 59 yr. old: 2.2 – 5.2 mmol/L (men) and 2.1 – 5.3 mmol/L (women)<br>60 – 64 yr. old: 2.2 – 5.1 mmol/L (men) and 2.3 – 5.3 mmol/L (women)<br>65 – 69 yr. old: 2.1 – 5.1 mmol/L (men) and 2.4 – 5.4 mmol/L (women)<br>70 – 74 yr. old: 2.1 – 5.1 mmol/L (men) and 2.3 – 5.3 mmol/L (women)<br>75 – 79 yr. old: 2.1 – 5.0 mmol/L (men) and 2.2 – 5.2 mmol/L (women)<br>≥80 yr. old: 2.1 – 4.7 mmol/L (men) and 2.3 – 5.3 mmol/L (women)
Liver function	
ALAT	<45 U/L (men) and <34 U/L (women)
ASAT	<35 U/L (men) and <31 U/L (women)
ALP	<115 U/L (men) and <98 U/L (women)
GGT	<55 U/L (men) and <38 U/L (women)
LDH	<248 U/L
Total bilirubin	<17 µmol/L
Kidney function	
Creatinine	65 – 115 µmol/L (men) and 55 – 90 µmol/L (women)
Albumin	35 – 50 g/L
Urea	2.5 – 7.5 mmol/L
eGFR	>60 ml/min before January 2021 and >90 ml/min after that date
Hematopoietic system	
Hb	8.6 – 10.5 mmol/L (men) and 7.5 – 9.5 mmol/L (women)
MCV	80 – 100 fL
RDW	12.0 – 16.0%
Electrolytes	
Na	136 – 145 mmol/L
K	3.5 – 5.1 mmol/L
Ca[a]	2.20 – 2.65 mmol/L

Abbreviations: ALAT, alanine aminotransferase; ALP, alkaline phosphatase; ASAT, aspartate transaminase; Ca, calcium; eGFR, estimated glomerular filtration rate; FSH, follicle stimulating hormone; fT4, free thyroxine; GGT, gamma-glutamyl transferase; Hb, hemoglobin; HbA1c, glycated hemoglobin; IGF-1, insulin-like growth factor 1; K, potassium; LDH, lactate dehydrogenase; LDL, low-density lipoprotein; LH, luteinizing hormone; MCV, mean corpuscular volume; Na, sodium; PTH, parathyroid hormone; RDW, red cell distribution width; SHBG, sex hormone-binding globulin; TSH, thyroid stimulating hormone

[a] Calcium corrected for albumin: measured calcium + 0.025 x (40 – albumin (g/L)) (1).

Table S2. Types of cardiac disorders

Type of cardiac disorder	Number of patients
Mitral insufficiency	4
Palpitations	3
Supraventricular tachycardia	1
Microvascular angina	1
Coronary artery disease	2
Aortic murmur	1
Decreased left ventricular function	1
Murmur II/IV	1
Heart failure (ischemic cardiomyophatie)	1

Table S3. Vitamin D deficiency and presence of neurofibromas

	Neurofibromas present (N = 103)	No neurofibromas present (N = 14)
Vitamin D deficiency	8	2
Vitamin D supplementation	68	8

Table S4. Laboratory measurements of 133 adults with Neurofibromatosis type 1

	All (N = 133)	Fatigue (N = 100)	No fatigue (N = 33)
fT4 (pmol/L)			
11 – 25 pmol/L			
Thyroid disorders included	17.7 (15.5 – 19.6)	17.1 (15.3 – 19.6)	18.6 (17.0 – 20.5)
Thyroid disorders excluded	17.1 (15.3 – 19.2)	16.9 (15.2 – 19.2)	18.0 (16.4 – 19.0)
13.5 – 24.3 pmol/L			
Thyroid disorders included	17.9 (16.7 – 20.4)	17.9 (16.5 – 20.2)	17.9 (16.9 – 21.7)
Thyroid disorders excluded	17.9 (16.9 – 20.3)	17.9 (16.6 – 20.2)	19.0 (16.9 – 21.5)
TSH (mU/L)			
0.4 – 4.3 mU/L			
Thyroid disorders included	1.51 (1.10 – 1.88)	1.53 (1.10 – 2.08)	1.33 (1.14 – 1.57)
Thyroid disorders excluded	1.51 (1.10 – 1.88)	1.68 (1.11 – 2.05)	1.28 (1.10 – 1.51)
0.56 – 4.27 mU/L			
Thyroid disorders included	1.68 (1.22 – 2.39)	1.68 (1.31 – 2.35)	1.53 (1.16 – 2.45)
Thyroid disorders excluded	1.68 (1.21 – 2.37)	1.70 (1.28 – 2.38)	1.27 (1.16 – 2.32)
Non-fasting glucose (mmol/L)			
Diabetes mellitus included	5.2 (4.9 – 5.7)	5.3 (4.9 – 5.6)	5.3 (4.9 – 5.9)
Diabetes mellitus excluded	5.2 (4.8 – 5.7)	5.3 (4.8 – 5.6)	5.3 (4.9 – 5.9)
25-OH-vitamin D (nmol/L)			
VitD supplementation included	57.0 (40.0 – 74.5)	58.0 (43.0 – 75.0)	54.0 (36.8 – 69.0)
VitD supplementation excluded	57.5 (41.8 – 75.8)	58.0 (38.0 – 75.0)	55.0 (53.0 – 92.0)
Creatinine (μmol/L)			
Male			
Low eGFR included	82.0 (72.0 – 89.3)	82.0 (72.0 – 88.0)	85.0 (80.0 – 89.0)
Low eGFR excluded	82.0 (72.0 – 86.5)	80.5 (71.3 – 85.3)	86.5 (81.5 – 89.5)
Female			
Low eGFR included	64.0 (59.0 – 71.0)	64.0 (58.0 – 71.5)	63.0 (59.0 – 69.0)
Low eGFR excluded	63.0 (59.0 – 70.0)	63.5 (58.0 – 70.0)	63.0 (60.5 – 68.0)
eGFR (ml/min)			
>60 ml/min			
low eGFR included	98.0 (89.8 – 114.5)	98.0 (90.0 – 116.5)	96.0 (88.0 – 113.0)
low eGFR excluded	98.0 (90.0 – 114.0)	99.0 (90.0 – 117.0)	95.5 (87.8 – 110.8)
>90 ml/min			
Low eGFR included	90.0 (90.0 – 106.0)	90.0 (90.0 – 106.0)	90.0 (81.0 – 108.0)
Low eGFR excluded	90.0 (90.0 – 108.0)	90.0 (90.0 – 106.0)	97.0 (86.5 – 108.5)
ALAT (U/L)			
Male			
Liver enzyme disturbances included	24.0 (19.0 – 31.0)	24.5 (19.0 – 31.3)	23.0 (19.0 – 30.0)

Liver enzyme disturbances excluded	23.0 (20.0 – 29.0)	23.5 (20.3 – 27.8)	23.0 (20.0 – 29.5)
Female			
Liver enzyme disturbances included	16.0 (13.0 – 21.0)	16.0 (13.0 – 21.5)	15.0 (14.0 – 19.0)
Liver enzyme disturbances excluded	15.0 (12.0 – 19.0)	15.0 (11.3 – 18.8)	15.0 (13.5 – 18.5)
ASAT (U/L)			
Male			
Liver enzyme disturbances included	21.0 (18.0 – 24.0)	20.5 (16.8 – 24.0)	22.0 (20.0 – 24.0)
Liver enzyme disturbances excluded	20.0 (19.0 – 24.0)	20.0 (18.3 – 21.0)	22.0 (20.0 – 24.0)
Female			
Liver enzyme disturbances included	17.5 (15.0 – 20.0)	17.0 (15.0 – 20.0)	18.0 (15.0 – 20.0)
Liver enzyme disturbances excluded	17.0 (14.0 – 19.0)	16.5 (14.0 – 19.0)	17.0 (14.0 – 18.5)
GGT (U/L)			
Male			
Liver enzyme disturbances included	34.0 (23.0 – 44.0)	32.5 (23.3 – 44.0)	37.0 (23.0 – 40.0)
Liver enzyme disturbances excluded	32.5 (24.0 – 39.0)	31.0 (24.0 – 39.0)	37.0 (24.0 – 39.0)
Female			
Liver enzyme disturbances included	20.5 (12.8 – 30.0)	21.0 (12.5 – 30.0)	17.0 (14.0 – 26.0)
Liver enzyme disturbances excluded	19.0 (12.5 – 26.0)	20.0 (12.5 – 26.5)	15.0 (13.3 – 25.3)
ALP (U/L)			
Male			
Liver enzyme disturbances included	67.5 (61.0 – 88.0)	66.0 (61.0 – 87.0)	68.0 (61.0 – 91.0)
Liver enzyme disturbances excluded	63.5 (59.0 – 69.8)	63.0 (59.0 – 69.0)	67.o (60.0 – 78.0)
Female			
Liver enzyme disturbances included	62.5 (54.0 – 77.8)	61.0 (54.0 – 76.8)	68.0 (53.8 – 78.0)
Liver enzyme disturbances excluded	60.5 (51.5 – 71.3)	59.0 (50.0 – 68.0)	65.0 (53.0 – 75.0)
LDH (U/L)			
Liver enzyme disturbances included	160.0 (137.0 – 182.0)	157.0 (135.0 – 182.0)	166.0 (153.5 – 180.8)
Liver enzyme disturbances excluded	154.0 (135.8 – 172.5)	149.0 (134.0 – 167.0)	168.0 (154.0 – 183.0)
Total bilirubin (μmol/L)			
Liver enzyme disturbances included	7.0 (5.0 – 9.0)	6.0 (5.3 – 8.0)	6.0 (6.0 – 8.0)
Liver enzyme disturbances excluded	6.0 (5.0 – 8.0)	6.0 (5.0 – 8.0)	7.0 (5.0 – 10.0)
Hb (mmol/L)			
Male			
Anemia included	9.7 (9.0 – 10.1)	9.7 (9.0 – 9.9)	9.4 (9.0 – 10.3)
Anemia excluded	9.7 (9.2 – 10.1)	9.8 (9.2 – 10.1)	9.7 (9.3 – 10.4)
Female			
Anemia included	8.6 (8.1 – 9.0)	8.6 (8.0 – 9.0)	8.5 (8.1 – 8.9)
Anemia excluded	8.7 (8.3 – 9.0)	8.8 (8.3 – 9.0)	8.6 (8.3 – 9.0)
MCV (fL)			
Anemia included	88.0 (84.0 – 90.0)	88.0 (84.0 – 91.0)	87.0 (85.0 – 88.0)

Anemia excluded	88.0 (85.0 – 90.5)	88.0 (85.5 – 91.0)	88.0 (85.0 – 88.5)
Potassium (mmol/L)	4.1 (3.9 – 4.3)	4.1 (3.9 – 4.3)	4.3 (4.0 – 4.4)
Sodium (mmol/L)			
Sodium abnormalities included	141.0 (139.0 – 142.0)	141.0 (139.8 – 142.0)	140.5 (139.0 – 142.0)
Sodium abnormalities excluded	141.0 (139.0 – 142.0)	141.0 (139.0 – 142.0)	141.0 (139.0 – 142.0)
Calcium[a] (mmol/L)			
Calcium abnormalities included	2.30 (2.23 – 2.36)	2.30 (2.22 – 2.37)	2.28 (2.25 – 2.34)
Calcium abnormalities excluded	2.32 (2.27 – 2.38)	2.33 (2.27 – 2.40)	2.30 (2.27 – 2.34)

Abbreviations: ALAT, alanine aminotransferase; ALP, alkaline phosphatase; ASAT, aspartate aminotransferase; eGFR, estimate glomerular filtration rate; fT4, free thyroxine 4; GGT, gamma-glutamyl transferase; Hb, hemoglobin; LDH, lactate dehydrogenase; MCV, mean corpuscular volume; TSH, thyroid stimulating hormone.
Data are displayed as median (IQR). During the study, the assay changed for fT4, TSH and eGFR. Therefore, different cut-off were used. More information about the reference values is given in the supplementary data. [a] Calcium corrected for albumin: measured calcium + 0.025 x (40 – albumin (g/L)) (1).
fT4 was missing in 15 adults (8 with and 7 without fatigue). TSH was missing in 15 adults (9 with and 7 without fatigue). Non-fasting glucose levels were missing in 18 adults (12 with and 6 without fatigue). 25-OH-vitamin D levels were missing in 11 adults (9 with and 2 without fatigue). Creatinine levels were missing in 10 adults (7 with and 3 without fatigue). eGFR levels were missing in 8 adults (3 with and 5 without fatigue). ALAT and ASAT levels were missing in 8 adults (5 with and 3 without fatigue). GGT levels were missing in 28 adults (19 with and 9 without fatigue). ALP levels were missing in 11 adults (7 with and 4 without fatigue). LDH levels were missing in 12 adults (7 with and 5 without fatigue). Total bilirubin levels were missing in 9 patients (5 with and 4 without fatigue). Hb levels were missing in 4 adults (1 with and 3 without fatigue). MCV levels were missing in 12 adults (8 with and 4 without fatigue). Potassium levels were missing in 8 adults (4 with and 4 without fatigue). Sodium levels were missing in 7 adults (4 with and 3 without fatigue). Calcium levels were missing in 17 adults (12 with and 5 without fatigue).

Table S5. Neurological examination in relation to fatigue in adults with Neurofibromatosis type 1

	**All (N = 53)**	**Fatigue (N = 37)**	**No fatigue (N = 16)**	**P-value**
Speech abnormalities	11 (33 missing)	9 (22 missing)	2 (11 missing)	0.69
Cranial nerve abnormalities	16 (11 missing)	12 (9 missing)	4 (2 missing)	0.46
Motor abnormalities	6 (11 missing)	4 (9 missing)	2 (2 missing)	0.65
Sensory abnormalities	1 (17 missing)	1 (13 missing)	0 (4 missing)	0.67
Coordination abnormalities	2 (16 missing)	0 (13 missing)	2 (3 missing)	0.07
Abnormal reflexes	2 (15 missing)	2 (11 missing)	0 (4 missing)	0.89
Abnormal gait	4 (16 missing)	3 (13 missing)	1 (3 missing)	0.52
Pain	41 (0 missing)	31 (0 missing)	10 (0 missing)	0.15

P-value was calculated with Fisher exact test because of low expected counts.

References

1. Cooper MS, Gittoes NJL. Diagnosis and management of hypocalcaemia. *Bmj*. 2008;336(7656):1298-1302.
